# A CBT-based mobile intervention as an adjunct treatment for adolescents with symptoms of depression: a virtual randomized controlled feasibility trial

**DOI:** 10.3389/fdgth.2023.1062471

**Published:** 2023-05-23

**Authors:** Vera N. Kulikov, Phoebe C. Crosthwaite, Shana A. Hall, Jessica E. Flannery, Gabriel S. Strauss, Elise M. Vierra, Xin L. Koepsell, Jessica I. Lake, Aarthi Padmanabhan

**Affiliations:** ^1^ Research Department, Limbix Health, San Francisco, CA, United States; ^2^ Science Department, Limbix Health, San Francisco, CA, United States; ^3^ Product Department, Limbix Health, San Francisco, CA, United States; ^4^ Content Department, Limbix Health, San Francisco, CA, United States

**Keywords:** cognitive behavioral therapy, digital therapeutics, adolescent depression, feasibility, mHealth, mental health

## Abstract

**Background:**

High rates of adolescent depression demand for more effective, accessible treatment options. A virtual randomized controlled trial was used to assess the feasibility and acceptability of a 5-week, self-guided, cognitive behavioral therapy (CBT)-based mobile application, Spark, compared to a psychoeducational mobile application (Active Control) as an adjunct treatment for adolescents with depression during the COVID-19 pandemic.

**Methods:**

A community sample aged 13–21, with self-reported symptoms of depression, was recruited nationwide. Participants were randomly assigned to use either Spark or Active Control (N_Spark_ = 35; N_Active Control_ = 25). Questionnaires, including the PHQ-8 measuring depression symptoms, completed before, during, and immediately following completion of the intervention, evaluated depressive symptoms, usability, engagement, and participant safety. App engagement data were also analyzed.

**Results:**

60 eligible adolescents (female = 47) were enrolled in 2 months. 35.6% of those expressing interest were consented and all enrolled. Study retention was high (85%). Spark users rated the app as usable (System Usability Scale_mean_ = 80.67) and engaging (User Engagement Scale-Short Form_mean_ = 3.62). Median daily use was 29%, and 23% completed all levels. There was a significant negative relationship between behavioral activations completed and change in PHQ-8. Efficacy analyses revealed a significant main effect of time, F = 40.60, *p* < .001, associated with decreased PHQ-8 scores over time. There was no significant Group × Time interaction (F = 0.13, *p* = .72) though the numeric decrease in PHQ-8 was greater for Spark (4.69 vs. 3.56). No serious adverse events or adverse device effects were reported for Spark users. Two serious adverse events reported in the Active Control group were addressed per our safety protocol.

**Conclusion:**

Recruitment, enrollment, and retention rates demonstrated study feasibility by being comparable or better than other mental health apps. Spark was highly acceptable relative to published norms. The study's novel safety protocol efficiently detected and managed adverse events. The lack of significant difference in depression symptom reduction between Spark and Active Control may be explained by study design and study design factors. Procedures established during this feasibility study will be leveraged for subsequent powered clinical trials evaluating app efficacy and safety.

**Clinical Trial Registration:**

https://clinicaltrials.gov/ct2/show/NCT04524598

## 1. Introduction

Depression, a highly prevalent mental health disorder among adolescents, is a growing crisis within the US (1, 2). Depressive episodes and symptoms affect up to 26% of adolescents annually, with depression and suicide rates rising sharply in recent years (1). Adolescent depression has far-reaching consequences including impairments in academic and work performance and social and family relationships, substance use, and exacerbation of other health conditions (3–6). Adolescent depression places significant economic burdens on the US healthcare system, with higher medical costs than those of almost any other adolescent mental health condition (7, 8). The COVID-19 pandemic disrupted the daily lives of adolescents around the globe, and it is estimated that global prevalence of depression symptoms amongst adolescents doubled as a result (9). With the demand for mental healthcare likely to continue increasing in coming years, the development of effective and accessible treatment options, such as digital interventions, is critical to reducing youth depression.

Despite high prevalence rates of depression, up to 80% of adolescents do not receive mental health treatment when necessary (10, 11). There are many reasons that adolescents do not receive adequate mental health care in times of need. First, social stigma surrounding mental healthcare causes adolescents to be hesitant to seek treatment (12). Additionally, limited access to effective mental health care means that those who do seek treatment are often unable to access it in times of need; because there is a nationwide lack of availability of speciality-trained clinicians, especially in rural areas, and mental health providers often get referrals from a variety of sources (primary care physicians, schools, self-referral) (13–15). Cost is also a barrier, with 11% of the population not seeking therapy because it is not covered by insurance, and an even bigger barrier for low-income individuals, with 30% of Medicaid patients reporting cost as an obstacle (16, 17). Finally, individuals who can afford treatment often do not have the time or ability to devote to weekly therapy, due to caregivers' employment commitments, school and after-school activities, or other responsibilities (18).

Digitally-delivered health interventions for mental illness address these barriers by providing private, accessible, cost-effective, and convenient means of treatment that can also increase engagement and self-disclosure due to lessened stigmatization (19–22). Critically, such interventions can serve as a first line of defense for treatment, eliminating wait times to access treatment and reducing high economic costs associated with traditional in-person psychotherapy. They are also available on demand so intervention sessions can be completed at the adolescent's convenience, and can be split into smaller sections of time, which may allow them to more readily fit into a daily routine. Digital treatments *via* mobile application hold particular promise as a widely-accessible treatment for adolescent mental illness– as adolescent smartphone ownership in the United States increased to 95% in 2018 (23). 45% of teens describe their internet use as “near constant” with around 9 in 10 teens reporting that they go online multiple times per day (23). The nearly universal use of smartphones within the U.S., which persists regardless of gender, race, ethnicity, and socioeconomic background, makes it a powerful tool to increase accessibility to mental health interventions (24). Therefore, digital technologies, such as mobile applications, could be leveraged to fill the depression treatment gap.

Cognitive-behavioral therapy (CBT) is a therapeutic approach that can be implemented in the context of digital therapeutics, which “deliver evidence-based therapeutic interventions that are driven by high quality software programs to prevent, manage, or treat a medical disorder or disease”(25). It is used for the prevention and treatment of depression in children and adolescents and is a recommended form of treatment by the American Academy of Pediatrics (26). Digital forms of CBT have been shown to be effective in the treatment of anxiety and depression in youth (27). Behavioral activation (BA), a core CBT skill that has been shown to be effective in conjunction with other CBT skills, like cognitive restructuring, or as a standalone treatment, is an activity performed so that the patient 1) increases engagement with adaptive and contextually relevant activities that induce feelings of mastery or pleasure, 2) advances their personal goals using a combination of motivational strategies, reward-seeking, natural reinforcers, and self-monitoring, and 3) reduces harmful and avoidant behaviors that often manifest during depressive episodes (28). BA-specific therapy is a successful method across multiple durations of treatment for treating depression in adolescents (29). Given that BA is individually paced, self-driven, and self-monitored, it can be easily delivered digitally, which may be appealing to depressed youth who have limited access to or lack of interest in traditional care. Recent evidence suggests that behavioral aspects of CBT are as effective as cognitive approaches in reducing depressive symptoms in youth and may mechanistically drive symptomatic reduction in CBT (30–32). A digital BA program for adolescent depression represents an exciting new direction for treatment. BA is a component of CBT treatment that emphasizes the connection between mood and behaviors. It has been shown to be successful when used in conjunction with other CBT skills, such as cognitive restructuring, but also when used as its own treatment, particularly for adolescents (33–36).

Digital applications of CBT are well supported as a comparable and effective alternative to traditional CBT (37). Computer-based CBT has been associated with significant effects on symptoms of depression in adolescents and growing evidence supports self-guided, smartphone based-apps as a promising treatment option for depression (38). While digital mental health interventions are an effective way to increase accessibility to proper mental health care, there remains a lack of digital treatment options for adolescents. To our knowledge, there are no digital therapeutics designed to treat adolescent depression approved by the FDA and the current study is the first feasibility trial for a digital therapeutic in adolescents. This digital BA program was designed to address the need for both accessible and evidence-based treatment for adolescents amidst a growing mental health crisis. The current research aimed to investigate the feasibility of a novel CBT-based mobile-app to treat adolescent depression.

This feasibility study was initiated during the COVID-19 pandemic as a means to provide accessible mental health resources to adolescents. The purpose of this randomized controlled trial (RCT) was to assess the feasibility and acceptability of a 5-week, self guided CBT-based mobile app program primarily focused on BA (Spark v2.0, hereafter referred to as Spark), compared to an active psychoeducational control condition (Active Control) for an adjunct treatment of adolescents with symptoms of depression. Study's primary aims included evaluating (1) study feasibility, based on recruitment rate, enrollment rate, and retention rate of participants, (2) acceptability of the app for the target population, based on usability (as evaluated by Systems Usability Scale [SUS] and post-intervention questionnaire responses) and engagement (as evaluated by the User Engagement Scale—Short Form [UES-SF]) and (3) the feasibility of a novel protocol for monitoring participant safety during a fully decentralized virtual clinical trial of a digital intervention, based on the rate of total number of clinical concerns identified in each group. A fourth (4) aim, considered a secondary aim, was to evaluate the preliminary evidence of clinical efficacy, exploring the differences in PHQ-8 score for each group over time, differences between groups in additional aspects of mood and health (Mood and Feelings Questionnaire [MFQ], Patient Reported Outcomes Measurement Information System—Pediatric [PROMIS—Pediatric], General Anxiety Disorder -7 [GAD-7] and Brief Resilience Scale [BRS]), and safety, determined by measuring the number of ADEs, SAEs, and UADEs identified in each group. The current study hypothesized that leveraging engaging mobile technologies would result in high treatment engagement, and preliminary evidence of clinical efficacy.

## 2. Materials and methods

### 2.1. Eligibility

Participants were eligible for the study if they 1) were between the ages of 13 and 21; 2) had self-reported symptoms of depression; 3) were residing in the USA for the duration of the 5-week study; 4) were under the care of a US-based primary care and/or licensed mental healthcare provider and willing to provide their provider's contact information (to contact them in case of a concern for participant safety); 5) were fluent and literate in English and had a legal guardian (if under 18 years of age) who was fluent and literate in English; 6) had access to an eligible smartphone (ie. one capable of downloading and running the digital therapeutic, meaning a iPhone 5s or later or running Android 4.4 KitKat or later); 7) had regular internet access (i.e., access to internet either within their home, school environment or other locations on a daily basis, with no planned time without regular internet access during the intervention period); and 8) were willing to provide informed e-consent/assent and had a legal guardian willing to provide informed e-consent (if under 18 years of age). The criteria that required participants to be under the care of a US based primary care and/or licensed mental healthcare provider was included to 1) evaluate the feasibility of the Spark app as an adjunct treatment for depression, and 2) to manage participant safety.

Participants were ineligible if they self-reported 1) a lifetime suicide attempt, 2) active self-harm, 3) active suicidal ideation with intent, or 4) a prior diagnosis by a clinician of bipolar disorder, substance use disorder, or any psychotic disorder including schizophrenia, or 5) if they were incapable of understanding or completing the study procedures or the digital intervention as determined by the participant, legal guardian, healthcare provider, or the clinical research team.

If participants were under the age of 18 and not determined to be legally emancipated, legal guardians were required to be involved in study procedures, including taking part in the initial onboarding session, providing consent, completing weekly questionnaires and receiving study correspondence when necessary.

Of note, the age range of 13–21 for study recruitment presents the variable adolescent period across individuals and is generally thought to extend through the second decade and into the third decade of life, roughly defined by the onset and completion of pubertal maturation as well as other psychosocial, socio-emotional, and cultural factors (39, 40). In the context of medical devices, including digital therapeutics, the US Food and Drug Administration (FDA) defines adolescence as between the ages of 12 and 21. Depression is also highly prevalent across this entire age range (41). As such, the goal of the current study was to assess feasibility of Spark as a digital therapeutic adjunct treatment for adolescent depression symptoms in this age range. We did not include those who were 12 years old due to Children's Online Privacy Protection Act (COPPA) restrictions for mobile applications in children under the age of 13.

### 2.2. Procedures

#### 2.2.1. Participant recruitment

Participants were recruited *via* online paid advertising on social media platforms, such Facebook and Instagram, and word of mouth. Paid advertisement campaigns were targeted towards 13–21 year-olds and the legal guardians of 13–17 year olds who were located within the US and English-speaking. After seeing and clicking on an advertisement, participants and/or legal guardians were directed to a landing page where they received an overview of the study and reviewed the presented eligibility criteria. If they determined themselves or their child eligible, they clicked on a link to schedule a consent appointment.

No formal power calculations were conducted to determine sample size. A target sample size of sixty was determined to be sufficient to evaluate feasibility, usability, and preliminary evidence of efficacy (42). This target sample size accounted for a predicted attrition rate of 20%–30% based on previous studies of digital CBT-based interventions for adolescent mental health (43–45). Recruitment was completed in two months, beginning July 23 2020, and ending on September 29 2020.

#### 2.2.2. Consent and Pre-intervention

This study was reviewed and approved by the Western Copernicus Group (WCG) Institutional Review Board (IRB) (ethical approval ID: WIRB® Protocol #20201686) with an abbreviated investigational device exemption for non-significant risk devices and was registered on clinicaltrials.gov (NCT04524598). This study was Phase I in two phases of clinical testing. In Phase II, a larger-scale RCT was conducted to evaluate the efficacy and safety of Spark, following product updates made as a result of Phase I study findings. These results will be reported elsewhere. The consent and onboarding process was completed *via* video conferencing, using the HIPAA-compliant Google Meet video-communication service, between a clinical research coordinator, the participant, and the participant's consenting guardian (if under 18). All participants provided written electronic informed consent, if over the age of 18, or assent, if under the age of 18. Written guardian informed consent was obtained from those under 18 years old.

After providing informed consent, participants and legal guardians were screened for eligibility, which involved the coordinator reviewing the criteria and the participant verbally confirming their eligibility. If the participant was under 18 years old, legal guardians were asked to leave the room while participants confirmed eligibility in order to provide the participant with a private setting to discuss sensitive topics, including self-harm and suicide/suicidal ideation. Afterwards, legal guardians returned to confirm their child's eligibility. Following the standard practice for health care providers, the research coordinators informed all participants about the limits of confidentiality, including the circumstances in which information related to safety risk would be shared with others. In clinical work with minors under the age of 18, these discussions involve what information will be shared with legal guardians. It is expected that information related to potential safety risk of minors would be shared with legal guardians so that appropriate services could be sought. We therefore expect a similar level of accuracy in reporting self-harm or suicide/suicidal ideation as what would occur in standard practice. Participants that met eligibility criteria during the onboarding session then used a web portal to fill out baseline questionnaires, including the Patient Health Questionnaire-8 (PHQ-8) (46), which measures symptoms of depression (see Questionnaires below). Baseline questionnaires took approximately 10–20 min to complete. Participants that met eligibility criteria were randomly assigned to the Spark or the Active Control group with a 1:1 ratio, using a fully random algorithm for randomization. Participants were guided by the coordinator to download the app and create an account. Once the participant logged in, they saw whether they had been randomized to Spark or the Active Control. Neither participants nor study staff were blinded to the assigned study condition. Participants and legal guardians were also provided with mental health resources and a safety plan (47) that could be completed in their own time.

#### 2.2.3. Five week intervention

Participants in both Spark and Active Control groups had access to their assigned app for a 5 week intervention period. All participants completed two weekly questionnaires in the app: 1) the PHQ-8 about their depression symptoms, and 2) an adverse events questionnaire (AEQ) about their safety (see Questionnaires below). These questionnaires took approximately 10–20 min to complete. Automated app notification reminders to complete these questionnaires were sent to participants. Legal guardians completed an AEQ on a weekly basis *via* a web portal. Both participants and legal guardians had access to their weekly questionnaires for seven days. Reminders were sent the day after the participant or legal guardian did not complete a weekly set of questionnaires, with a warning that participants would be withdrawn if they did not complete the AEQ questionnaire due to being unable to monitor their safety. If a participant did not complete the weekly questionnaires two weeks in a row, they were emailed that they will be withdrawn from the study. Both emails were templated.

##### 2.2.3.1. Spark group

**Figure 1:**
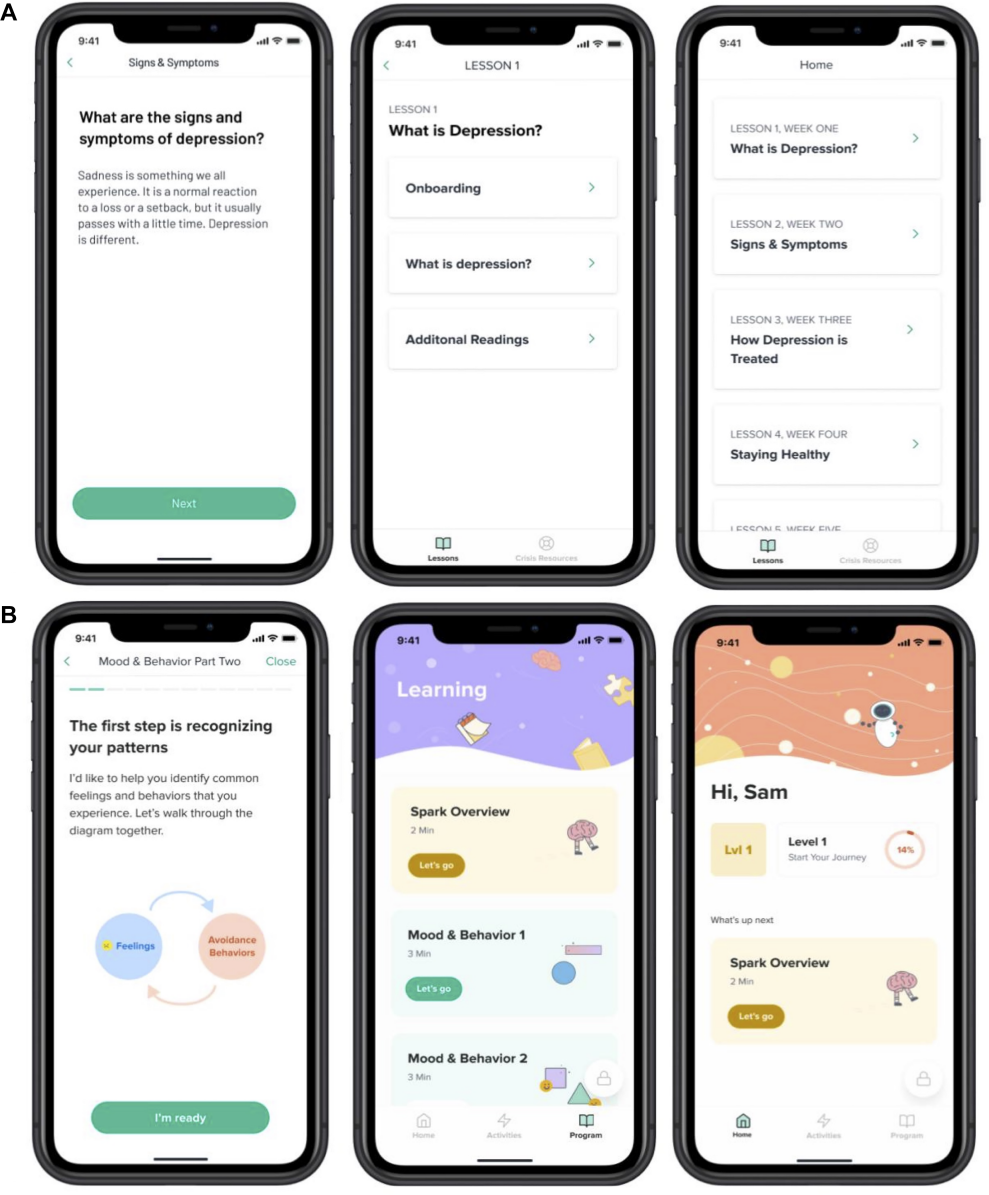
Examples of screens from the Active Control (**A**) and Spark (**B**) apps.

The treatment intervention, Spark (v2.0), was a 5-level, interactive program. Our program was modeled on evidenced based treatment (EBT) protocols for behavioral activation (35, 48–52), particularly for adolescents. Following those EBTs, we retained the same therapeutic ingredients: 1) an introduction to the BA model 2) getting active and charting progress (including focus on BAs, tracking mood and behavior, and identifying activities that align with users values), 3) skill building and addressing barriers and avoidance (includes sessions on problem solving, goal setting, and identifying barriers that can get in the way of accomplishing goals), 4) practice (includes practice and consolidation of skills), 5) moving forward/planning for continued activation (includes review of treatment gains, and relapse prevention strategies). This version of our intervention built upon the previous version of the app called Spark (v1.0) (53). User experience data from post-study interviews, from a previous study of an earlier version of Spark, was used to inform the design of the version of the Spark app used in this study. Levels in the app progress in a linear fashion; participants had to complete each task before they could progress onto the next task. Each level was designed to take less than 60 min and participants were recommended to complete one level per week, though they could progress at their own pace. Participants were guided through the program by a character called “Limbot.” This character encourages the user to complete the program and provides personal examples of how they have undertaken behavioral activation therapy. In level 1, participants completed onboarding and learning tasks. During onboarding, they received a tutorial on the app interface and a description of the BA program. The first learning task included information about the behavioral (BA) model of depression, focusing on the relationship between mood and behavior, and how it can lead to a downward cycle of depression. Next, participants learned about breaking the cycle of depression by changing behavior. They received information about how completing activities that align with their values can help the activities be more effective at improving their mood. Participants identified values that were important to them (54). At the end of lesson 1, participants were taught how to schedule activities centered around their previously identified values and were given a walkthrough tutorial of the activities tab. Level 2 through Level 5 focused on activity scheduling and review. Participants were asked to schedule activities within the app and then complete those activities outside of the app. Participants were encouraged to log into the app and reflect on the activity that they completed, answering questions about how the activity aligned with their selected values (Lesson 1) and how it made them feel. If participants did not complete their scheduled activity, they were asked questions that encouraged them to reflect upon the roadblocks they encountered and how they can combat them in the future. At the end of each level, participants received acknowledgement from the Limbot character and learned about the goal for the next level. Crisis resources could be accessed in the app at any time. See Figure 1 for an illustration of the app interface.

##### 2.2.3.2. Active control group

The Active Control was an app containing educational content related to symptoms and treatments for depression, healthy habits and resources. The content was largely based on the NIMH Teenage Depression ebook (55). It did not include CBT or BA components. Participants did not have the ability to enter free form text in the app. The Active Control was designed to be similar to Spark in duration, and modality of delivery and contained five lessons. Content in the Active Control app was not gated; it was possible to access later lessons without having reviewed earlier lessons. See Figure 1 for an illustration of the interface.

#### 2.2.4. Post-Intervention

After the 5-week intervention period, participants and their legal guardian were emailed links to complete post-intervention self-report assessments, which took approximately 10–20 min to complete. Participants and their legal guardian received reminders to complete their assessments if they did not complete the questionnaires after one week of being granted access. These emails were templated. Participants who did not complete the post-intervention assessments within 4 weeks from the end of the intervention period lost access to their assessments at that time and were considered lost to follow up. Participants were compensated $25 in the form of an electronic gift card for completing the post-intervention assessments regardless of app usage.

#### 2.2.5. Post-Intervention interviews

Select participants and legal guardians were invited to participate in 1 hour interviews for product feedback. Participants were selected to take part in these interviews based on different factors including age, geographic location, and level of app engagement. Participants were compensated $25 in the form of an electronic gift card for participating. These data are out of scope for this manuscript and are not discussed further.

### 2.3. Safety protocol

During the study period, trained study staff followed a rigorous safety protocol with study PI and clinician oversight. Clinical concerns that arose at any time during the study were logged. Clinical concerns were defined as any potentially concerning information reported during the trial that indicated a potential risk to health in the past, present, or future, or that signaled abuse. Clinical concerns were identified through four channels:

• Text entered within the Spark app identified by a research coordinator as concerning (defined by the safety protocol)
• Deterioration of symptoms of depression, defined as a PHQ-8 score ≥ 15 (moderately severe or higher) (46) and a ≥ 5 point increase from baseline (56)
• Text in any questionnaire identified by a research coordinator as concerning
• Spontaneously reported harm by participants or legal guardians, including self-harm or abuse, during direct communication with study staff or *via* email

Any clinical concern identified during the study triggered the safety protocol, regardless of severity. The safety protocol dictated that, during the onboarding session, if a participant indicated that they were in immediate distress or danger, the study coordinator would direct them towards emergency services (e.g., the nearest emergency room or calling 911). Otherwise if a clinical concern was identified in an asynchronous context, or during the onboarding session but did not require immediate referral to emergency services, it was escalated to the study investigator. Study investigators reviewed mild concerns weekly and moderate concerns within 24 h, along with any other relevant information or safety data. The study investigator would determine whether the clinical concern required escalation to the study clinician based on criteria established in the safety protocol and within 48 h the study investigator would determine whether the participant was safe and eligible to continue with the study, consulting with the study clinician as needed. If the safety concern was related to suicidality, the study investigator or clinician was trained to administer the Ask Suicide-Screening Questions (ASQ) toolkit (57). If the study clinician determined that the participant was no longer eligible to continue with the study, or if the clinician could not monitor safety due to not being able to reach the participant or other listed contacts, the participant would be informed, withdrawn from the study, and sent mental health resources. Participants were also withdrawn from the study if they did not complete the weekly Adverse Event Questionnaire for two consecutive weeks. (Note: this procedure was implemented in the second month of enrollment, as during this virtual and decentralized RCT we were otherwise unable to determine participant safety).

After study completion, an internal clinician who was not otherwise involved in the study, reviewed all clinical concern data. Those that the clinician judged to be potential adverse events were sent to an external clinician. These clinical concerns, along with accompanying relevant safety data, were classified as relevant as adverse events (AE), adverse device effects (ADE), serious adverse events (SAE), and unanticipated adverse device effects (UADE) (58–60). Definitions used for adverse events classification can be found in Table 1.

**Table 1:** Definitions for external clinician categorization of adverse events (AEs).

Adverse Event	**An adverse event (AE)** is an untoward medical occurrence, unintended disease or injury, or untoward clinical signs (including abnormal laboratory findings) in subjects (3.50), users or other persons, whether or not related to the investigational medical device (3.29) and whether anticipated or unanticipated. Note 1 to entry: This definition includes events related to the investigational medical device or the comparator (3.12). Note 2 to entry: This definition includes events related to the procedures involved.
Serious Adverse Event	**Serious Adverse Events/Serious Adverse Device Effects**: An adverse event or adverse device effect is considered serious if it meets any of the following criteria: • Is fatal; • Is life-threatening, meaning, the participant was, in the view of the investigator, at immediate risk of death from the reaction as it occurred; • Leads to persistent or significant disability/incapacity, i.e., the event causes a substantial disruption of a person's ability to conduct normal life functions; • Requires or prolongs inpatient hospitalization; • Is an important medical event, based on appropriate medical judgment, that may jeopardize the participant, or the participant may require medical or surgical intervention to prevent one of the other outcomes above. Note 1: Planned hospitalization for a pre-existing condition, or a procedure required by the CIP (3.9), without serious deterioration in health, is not considered a serious adverse event. Note 2: Serious adverse device effect (SADE): adverse device effect that has resulted in any of the consequences characteristic of a serious adverse event.
Adverse Device Effect	**An adverse device effect (ADE)** is an adverse event related to the use of an investigational medical device. This includes any adverse event resulting from insufficiencies or inadequacies in the instructions for use, the deployment, the implantation, the installation, the operation, or any malfunction of the investigational medical device. This also includes any event that is a result of a user error or intentional misuse. Note: For this study, ADEs may occur in either the Spark or Active Control arms.
Unanticipated Adverse Device Effect	(UADEs, as defined in 21 CFR 812.3, also referred to as “Unanticipated Problems”): Any serious adverse effect on health or safety or any life-threatening problem or death caused by, or associated with, a device, if that effect, problem, or death was not previously identified in nature, severity, or degree of incidence in the investigational plan or application; OR Any other unanticipated serious problem associated with a device that relates to the rights, safety, or welfare of subjects.

### 2.4. Questionnaires

Different measures were used to assess the characteristics of the study population, general mood, depression and anxiety symptoms, and overall health. All questionnaires were delivered to both Spark and Active Control users. The schedule of assessments can be referenced in Table 2.

**Table 2:** Baseline and post-intervention assessments for participants and legal guardians were completed *via* a secure web portal. Weekly participant assessments were completed in the mobile app. Weekly parent assessments were completed *via* a secure web portal.

	Baseline	Weekly during the 5-week intervention	Post- intervention
Patient Health Questionnaire (PHQ-8)*	X	X	X
Baseline Questionnaire- Participant*	X		
Baseline Questionnaire- Parent*	X		
Brief Resilience Scale (BRS)	X		
Generalized Anxiety Disorder (GAD-7)*	X		X
PROMIS Pediatric Global Health Scale*	X		X
PROMIS Parent Proxy Global Health Scale	X		X
Mood and Feelings Questionnaire (Short Parent Version)*	X		X
Adverse Events Questionnaire- Participant*		X	X
Adverse Events Questionnaire- Parent*		X	X
Post-intervention Questionnaire- Participant*			X
Post-intervention Questionnaire- Parent*			X
System Usability Scale*			X
User Engagement Scale—Short Form*			X

*Indicates Questionnaires that were reported in this manuscript.

#### 2.4.1. Baseline demographics questionnaire

The Baseline Demographics Questionnaire was an internally developed questionnaire that included demographic questions in regards to the adolescent participant's gender (i.e., male, female, or gender non-binary), ethnicity, race, and age, questions about prior and current treatment for depression and other mental health disorders. Choice questions, with answer choices of “yes” or “no” were used to evaluate whether the participant had been diagnosed with depression or any other mental health, cognitive, or developmental disorder, followed by a free-form text field asking for details about any disorder, besides depression, with which they had been diagnosed. A multi-select choice question was used to evaluate previous or concurrent treatment for depression, with a free-form text field provided if the participant selected “Other” for forms of treatment. A free-form text field was also provided, asking the participant to list all medication they were taking when beginning the intervention. Separate versions of the baseline demographics questionnaire were completed by participants and legal guardians, where legal guardians completed questions about their education level, and their child's demographics, diagnosis and treatment.

#### 2.4.2. Patient health questionnaire (PHQ-8)

The PHQ-8 consists of eight descriptive phrases of depressive symptoms (61). Participants rated how often they were bothered by any of those symptoms over the last fortnight; (0) Not at All; (1) Several Days; (2) More than Half the Days; (3) Nearly Every Day. Possible scores ranged from 0 to 24, with a higher score indicating more severe depressive symptoms. This assessment was delivered at baseline, weekly during the 5-week intervention and post-intervention. Only the participant completed the PHQ-8. Participants had a full week to complete each weekly PHQ-8 in app after the baseline PHQ-8. Participants had one month to complete the post-intervention PHQ-8. The PHQ-8 is a well established measure to both diagnose and assess the severity of depressive disorders (62). Evidence supports the high internal reliability of the PHQ-8 (Cronbach's *α* = .89) and its high construct validity, with the PHQ-8 score correlating strongly with patient mental health (.73) (46).

#### 2.4.3. Adverse event questionnaire (AEQ)

The AEQ was an internally developed questionnaire that assessed consenting guardian- and participant-reported clinical concerns. Participants and legal guardians were asked to rate clinical concerns in terms of severity, on a scale of (0) Not at all to (4) Extremely, to provide the start and stop date (if applicable), and to indicate whether they believed the reported concern was related to study intervention. This assessment was delivered during the 5-week intervention and at post-intervention. Separate versions of the AEQ were completed by the participant and legal guardian.

#### 2.4.4. Post-intervention questionnaire

The post-intervention questionnaire was developed internally and administered at post-intervention including questions about current treatment for depression and other mental health disorders and any changes in treatment since baseline. The questionnaire also asked whether participants and legal guardians thought the program helped them, and questions evaluating participant experience using the program as a whole. Mood improvement was captured through the following question for participants: “How much do you feel like this mobile app improved your symptoms of depression?” and for parents: “How much do you feel like this mobile app improved your child's symptoms of depression?”. Respondents indicated their response using a 10 point scale (0 = Didn't improve at all, 5 = Moderately Improved, 10 = Improved Completely). Participants and legal guardians completed different versions of the post-intervention questionnaire.

#### 2.4.5. The system usability scale (SUS)

The SUS is a validated scale used to assess the usability of a system originally developed by Brooke (63). It was modified for use in this study to evaluate app usability at post-intervention. It consisted of 10 questions about how easy it was to use the app (63, 64). Responses are given on a 5-point Likert scale from (0) Strongly Disagree to (4) Strongly Agree. Item responses are summed and multiplied by 2.5 such that final scores range from 0 to 100. A score above 68 is considered above average. Only the participant completed the SUS. The SUS is supported as an easy to administer yet highly reliable method (Cronbach's *α* = 0.911) for measuring the usability of a product (65).

#### 2.4.6. The user engagement scale short form (UES-Sf)

The UES-SF has 12 questions about how engaging participants found the app (66) and was delivered post-intervention. Responses are given on a 5-point Likert scale from (1) Strongly Disagree to (5) Strongly Agree. Item responses are averaged across all questions to generate a general engagement score ranging from 1 to 5. Only the participant completed the UES-SF. Data supports the UES-SF as a statistically reliable scale that can effectively estimate full UES scores (66).

#### 2.4.7. Generalized anxiety disorder 7-item scale (GAD-7)

The GAD-7 is a brief seven-item self-report measure of anxiety. The scale has been found to be reliable and valid (67), and was used to evaluate changes in anxiety given the high comorbidity between anxiety and depression. The GAD-7 scale was delivered at baseline and post-intervention. This assessment was delivered at baseline and post-intervention. Only the participant completed the GAD-7.

#### 2.4.8. PROMIS pediatric global health scale & PROMIS parent proxy global health scale

These are 9-item measures that produce essentially a unidimensional measure of global health perception/well-being³. The PROMIS Parent Proxy Global Health Scale was written parallel to the PROMIS Pediatric Global Health Scale to allow consenting guardians to report on the perceived global health/well-being of their child. These scales are supported as a brief and reliable method to measure the global health status of children (68, 69). Both scales start with 4 descriptive phrases paired with scale of 5–1, asking the user to evaluate different aspects of their global health perception/well-being; (5) Excellent, (4) Very Good, (3) Good, (2) Fair, (1) Poor, followed by 3 questions with descriptive phrases paired with a scale of 5–1; (5) Always, (4) Often, (3) Sometimes, (2) Rarely, (1) Never; and two final phrases paired with a scale of 1–5, (1) Never, (2) Almost Never, (3) Sometimes, (4) Often, (5) Almost Always. Possible scores ranged from 0 to 24, with a higher score indicating a lower quality of life. The PROMIS scales were delivered at baseline and post-intervention. The consenting guardian completed the PROMIS Parent Proxy Global Health Scale 7 + 2 and the participant completed the PROMIS Pediatric Global Health Scale.

#### 2.4.9. Mood and feelings questionnaire short parent version (MFQ-Ps)

The MFQ-PS was used to record change in parent-reported depressive symptoms. The MFQ consists of 13 descriptive phrases paired with scales rated 0–2; (0) True, (1) Sometimes, (2) Not True. Possible scores range from 0 to 26, with a higher the score indicating the higher the likelihood the child is suffering from depression, as reported by a consenting guardian. The MFQ-PS was delivered at the baseline and post-intervention. Only the consenting guardian completed the MFQ-PS. This scale is supported as a brief and reliable method of evaluating depressive symptoms (70).

#### 2.4.10. Brief resilience scale (BRS)

The BRS is a 6 item self-report measure for assessing the ability to “bounce back” or recover from stress. It has been shown to be reliable and to measure a unitary construct (71). The BRS was delivered at the baseline. Only the participant completed the BRS.

A description of an additional exploratory questionnaire (COVID questionnaire) administered during the study can be found in the Supplementary Materials.

### 2.5. Analysis

#### 2.5.1. Participant characteristics and feasibility outcomes

Participant characteristics were evaluated per study arm and for the full study sample. Chi-squared tests and two-sample *t*-tests were used to evaluate significance of any group differences, as appropriate. Study feasibility was evaluated as 1) recruitment rate: the proportion of those who scheduled an onboarding session out of those who expressed interest in the study, 2) enrollment rate: the proportion of participants enrolled in the study out of those who scheduled an onboarding session and 3) retention rate: the proportion of those who completed the post-intervention survey out of those who enrolled in the study. Microsoft Excel was used to analyze these data.

#### 2.5.2. App acceptability: usability and engagement

App acceptability consists of app usability and engagement. Usability was collected *via* the SUS and post-intervention questionnaire. Exploratory comparisons of SUS, post-intervention questionnaire, and UES-SF scores were conducted between the Spark and Active Control groups using two-sample *t*-tests. Spark app engagement was collected *via* self-report, the UES-SF, and app usage data. App usage data included: (1) the percent of daily active users who used Spark on each intervention day, along with the median percent of daily active users across the full intervention period; and (2) the percent of Spark participants who completed each of the five levels of Spark, along with the percent of participants who completed behavior activation activities. Daily active use was defined as opening the app for any duration. Descriptive statistics are reported for app usage. Finally, a correlation was run to examine the relationship between post-intervention and baseline PHQ-8 scores and the number of behavioral activation activities completed. Microsoft Excel was used to analyze these data.

#### 2.5.3. Study safety protocol feasibility

The number of total clinical concerns identified in each group was evaluated. We used free-form text to identify clinical concerns in the Spark group. We note that the Active Control group did not have the ability to enter free-form text into the app. Therefore, we report descriptive statistics about the total number of clinical concerns captured for each group without direct comparison. We report the sources of clinical concerns, the number of participants that had clinical concerns escalated to the study clinician, and the number of participants that had clinical concerns that elicited clinician reachout to the participant. The feasibility of capturing clinical concerns through a variety of sources and of managing safety concerns in a fully virtual setting was evaluated. Microsoft Excel was used to analyze these data.

#### 2.5.4. App efficacy and safety

Differences in PHQ-8 scores for each group over time were explored. Multiple imputation was used to account for missing data points, excluding participants with only baseline scores. First, analyses were conducted to determine if data were missing completely at random and whether patterns of missing data differed between groups. Little's test (72) was used to determine whether data were missing completely at random and a chi-square test was conducted to identify whether there were significant differences between groups in the proportion of missing data across weeks. Because participants had seven days to complete each weekly PHQ-8, the assumption that spacing between the six timepoints was consistent across time and groups was evaluated using a generalized linear mixed-effect model (GLMM) with a 2-level PAN method (73) with numbers of days since baseline PHQ-8 completion as the dependent variable. Main effects of Group (Treatment vs. Active Control) and Week (six timepoints) were analyzed along with the Group × Week interaction. Finally, to test for group differences in the change in PHQ-8 scores over time,an exploratory GLMM was conducted using a 2-level PAN method and examined the main effects of Group, Week, and the Group × Week interaction. Days between successive PHQ-8 completions was included as a random-effect for the slope at the individual level to control for irregular spacing between questionnaire completion timepoints. Random effects also included a participant-level intercept. As the primary objective of this study was not to evaluate efficacy, this analysis was not powered to detect significant group differences in PHQ-8 scores. An exploratory analysis measured the change in PHQ-8 scores between baseline and post-intervention for individuals with a baseline PHQ-8 score ≥ 10, consistent with moderate symptoms of depression in both groups. Descriptive statistics are presented for this analysis. R version 4.1.1 (2021–08–10) was used to complete these analyses, and included using self-written code and the following packages: Rmisc, reshape2, stringr, ggplot2 and lmerTest.

The standardized mean-difference effect size and 95% confidence intervals were calculated for the MFQ, PROMIS Pediatric, GAD-7, and BRS measures using the Practical Meta-Analysis Effect Size Calculator created by W. Lipsey and David B. Wilson, 2001.

App safety was determined by measuring the number of ADEs, SAEs, and UADEs identified in each group. Descriptive statistics about the number of AEs, ADEs, and UADEs captured for each group are reported. Microsoft Excel was used to analyze these data.

## 3. Results

### 3.1. Participant characteristics & feasibility outcomes

**Figure 2:**
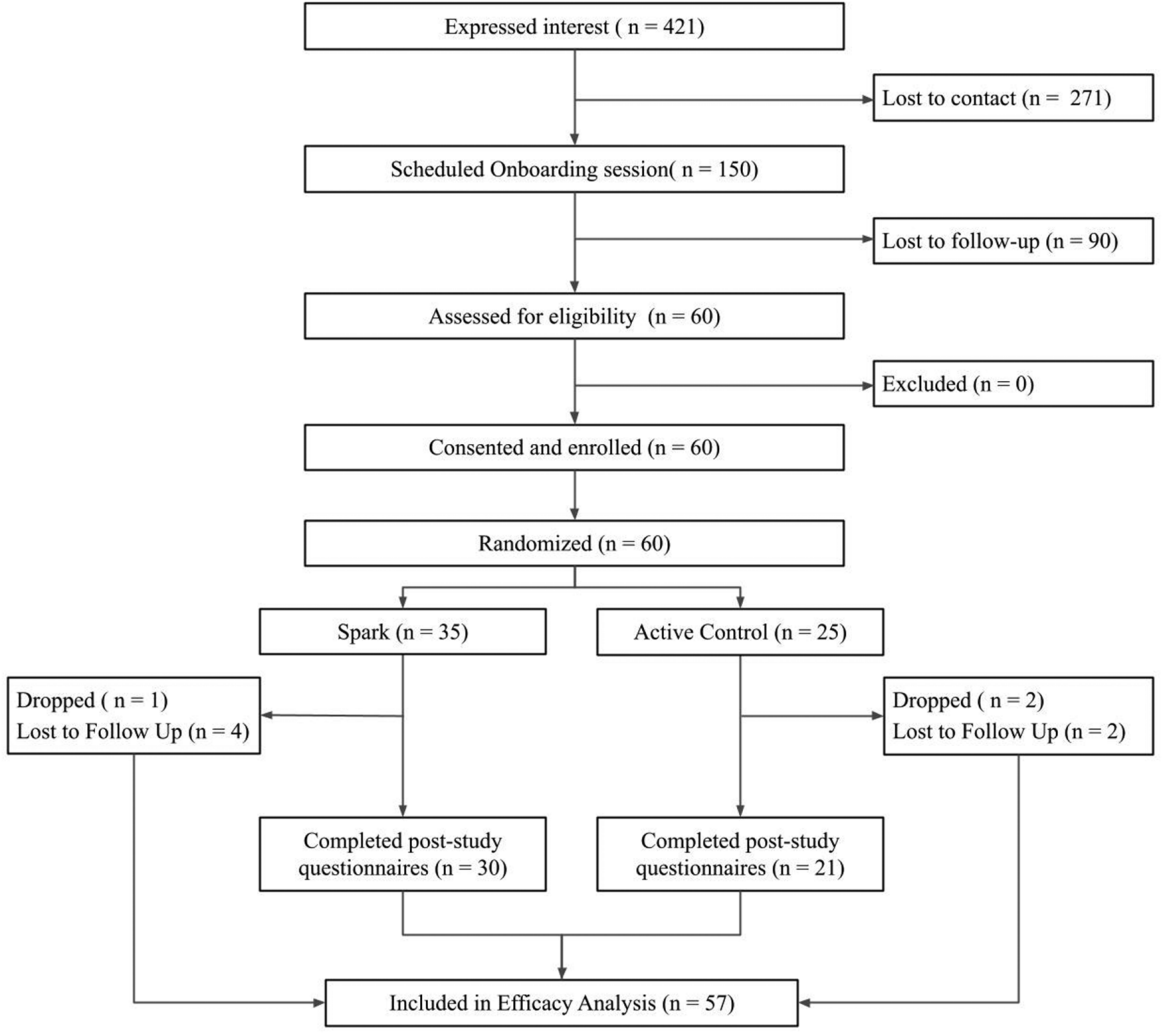
The flow of participants through the study procedures, from expression of interest to efficacy analysis.

Over two months, sixty eligible participants were enrolled in the study. See Figure 2 for the CONSORT diagram. 421 participants expressed interest in the study *via* a web form, of which 150 scheduled an onboarding session, representing a 35.6% recruitment rate. Of the 150 who scheduled an onboarded session, 60 attended their onboarding session, were determined to be eligible to participate, consented/assented and were enrolled, representing a 40% enrollment rate. Of these 60 participants, 35 were randomized to the Spark arm and 25 to the Active Control arm. 51 participants completed the study (n_Spark _= 30, n_Active Control_ = 21), representing a 85% retention rate by post-intervention. Of those that did not complete the study, 3 participants (n_Spark _= 1, n_Active Control_ = 2) were withdrawn per the safety protocol, due to missing two consecutive weekly questionnaires or safety events, and 6 participants were considered lost-to-follow up (n_Spark _= 4, n_Active Control _= 2) due to not completing post-intervention questionnaires.

See Table 3 for participant characteristics. The sample recruited, consisting of 13–21 year olds (n_Spark_ = 17.91 [2.36]; n_Active Control_ = 16.96 [2.57]),was 78% female, which is consistent with higher rates of depression in adolescent girls (74, 75). The average PHQ score at baseline was 13.82, which is considered moderate severity (46). The majority of participants (*n* = 32, 53%) reported a depression diagnosis and 28 participants (46.6%) reported that they were currently receiving treatment specifically for depression at baseline. The majority of participants (*n* = 37, 62%) were over 18 years old in both conditions (n_Spark_ = 19; n_Active Control_ = 18). Additionally, 29 legal guardians (n_Spark_ = 16; n_Active Control_ = 13) were enrolled.

**Table 3:** Baseline characteristics of adolescent participants and legal guardians enrolled within the study.

Adolescent Participants			
	Spark (*N* = 35)	Active Control (*N* = 25)	Test Statistic
Age, M (SD)	17.91 (2.36)	16.96 (2.57)	*t*(*58*) = 2.00, *p* = .14
Gender, *N* (%)			*χ^2^ (2)* = .93, *p* = .62
*Male*	6 (17.14%)	5 (20.00%)	
*Female*	28 (80.00%)	19 (76.00%)	
*Non-binary*	1 (2.86%)	1 (4.00%)	
Race, *N* (%)			*χ^2^ (5)* = .59, *p* = .99
*American Indian/Alaska Native*	1 (2.86%)	0 (0.00%)	
*Asian*	7 (20.00%)	4 (16.00%)	
*Black or African American*	2 (5.71%)	3 (12.00%)	
*Native Hawaiian or Other Pacific Islander*	0 (0.00%)	0 (0.00%)	
*Unknown*	2 (5.71%)	0 (0.00%)	
*White*	20 (57.14%)	17 (68.00%)	
*Mixed Race*	3 (8.57%)	1 (4.00%)	
Ethnicity, *N* (%)			*χ^2^ (1)* = .91, *p* = .34
*Hispanic/Latino*	6 (17.14%)	4 (16.00%)	* *
*Not Hispanic/Latino*	29 (82.85%)	21 (84.00%)	* *
Baseline PHQ-8, M (SD)	13.74 (6.02)	13.92 (5.32)	*t(58)* = 2.00, *p* = .90
Severity, *N* (%)			*χ^2^ (1)* = .86, *p* = .35
*mild-moderate (up to 15)*	23 (65.71%)	16 (64.00%)	
*moderate to severe (above 15)*	12 (34.29%)	9 (36.00%)	
Depression Diagnosis, *N* (%)	18 (51.43%)	14 (56.00%)	*χ^2^ (1)* = .73, *p* = .39
Concurrent treatment for depression, *N* (%)			*χ^2^ (5)* = .57, *p* = .99
*Medication only*	5 (14.29%)	8 (32.00%)	
*None*	19 (54.29%)	12 (48.00%)	
*Other*	1 (2.86%)	0 (0.00%)	
*Psychotherapy only*	4 (11.43%)	2 (8.00%)	
*Medication and Psychotherapy*	5 (14.28%)	3 (12.00%)	
*Unknown*	1 (2.86%)	0 (0.00%)	
Legal Guardians			
* *	Spark (*N* = 16)	Active Control (*N* = 13)	
Education Level, *N* (%)			*χ^2^ (5)* = .50, *p* = 0.99
*Middle school*	3 (18.75%)	1 (7.69%)	
*High school/GED*	1 (6.25%)	0 (0.00%)	
*Some college*	1 (6.25%)	3 (23.07%)	
*Associate's and/or Bachelor's degree*	9 (56.25%)	6 (46.15%)	
*Master's degree*	2 (12.50%)	2 (15.38%)	
*Doctoral or Professional degree*	0 (0.00%)	1 (7.69%)	

### 3.2. App acceptability: engagement & usability

As seen in Table 4, participants reported using Spark to be a more engaging experience than using the Active Control on the UES-SF (*t*(49) = 3.46, *p* < .005). Both apps were rated as having above-average usability, as indicated by a score of 68 or higher on the SUS scale. Exploratory between-group analyses were conducted. No differences were found as measured by the SUS mean scores in each condition (*t*(49) = 1.50, *p* > .1). Additionally, participants that used Spark reported a higher average improvement in symptoms of depression than participants that used the Active Control (*t*(49) = 4.96, *p* < .001). Legal guardians of participants who used either Spark or the Active control did not indicate a difference in subjective reports of symptom improvement between the two apps (*t*(16) = 0.83, *p* > .1). Both participants who used Spark and the legal guardians of these participants reported higher enjoyability ratings of the app compared to the Active Control users (participants: *t*(49) = 4.55, *p* < .001) and their legal guardians: *t*(16) = 2.77, *p* < .05). See Table 5 for more detail.

**Table 4:** The mean SUS and UES-SF scores for the two conditions. The mean usability and engagement for Spark users was higher than for the Active Control.

	Spark (*N* = 30)	Active Control (*N* = 21)	Test Statistic
SUS, M (SD)	80.67 (11.91)	75.83 (10.50)	*t(49)* = 1.50, *p* = .14
UES-SF, M (SD)	3.62 (0.52)	3.10 (0.54)	*t(49)* = 3.46, *p* = .001

**Table 5:** Post-intervention questionnaire app feedback question ratings.

Question	Participants (*n* = 51)			Parents (*n* = 18)		
Question (on a scale of 0–10)	Spark (*n* = 30), Mean (SD)	Active Control (*n* = 21), Mean (SD)	*t*-test	Spark (*n* = 10), Mean (SD)	Active Control (*n* = 8), Mean (SD)	*t*-test
Mood improvement	5.07 (2.30)	1.90 (2.17)	*t(49)* = 4.96, *p* < .001	4.90 (1.91)	2.88 (3.27)	*t(16)* = 0.83, *p* > .1
Enjoyableness of mobile app	6.83 (2.05)	3.95 (2.46)	*t(49)* = 4.55, *p* < .001	6.10 (1.85)	2.75 (3.24)	*t*(*16)* = 2.77, *p* < .05

**Figure 3:**
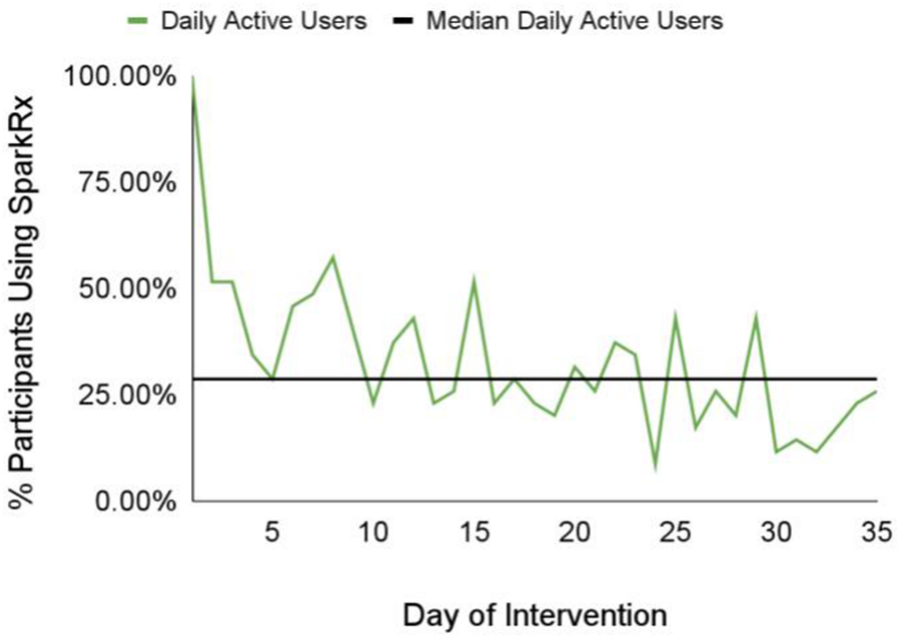
The median value and daily number of daily active users on a given day across the 5-week intervention period.

**Figure 4:**
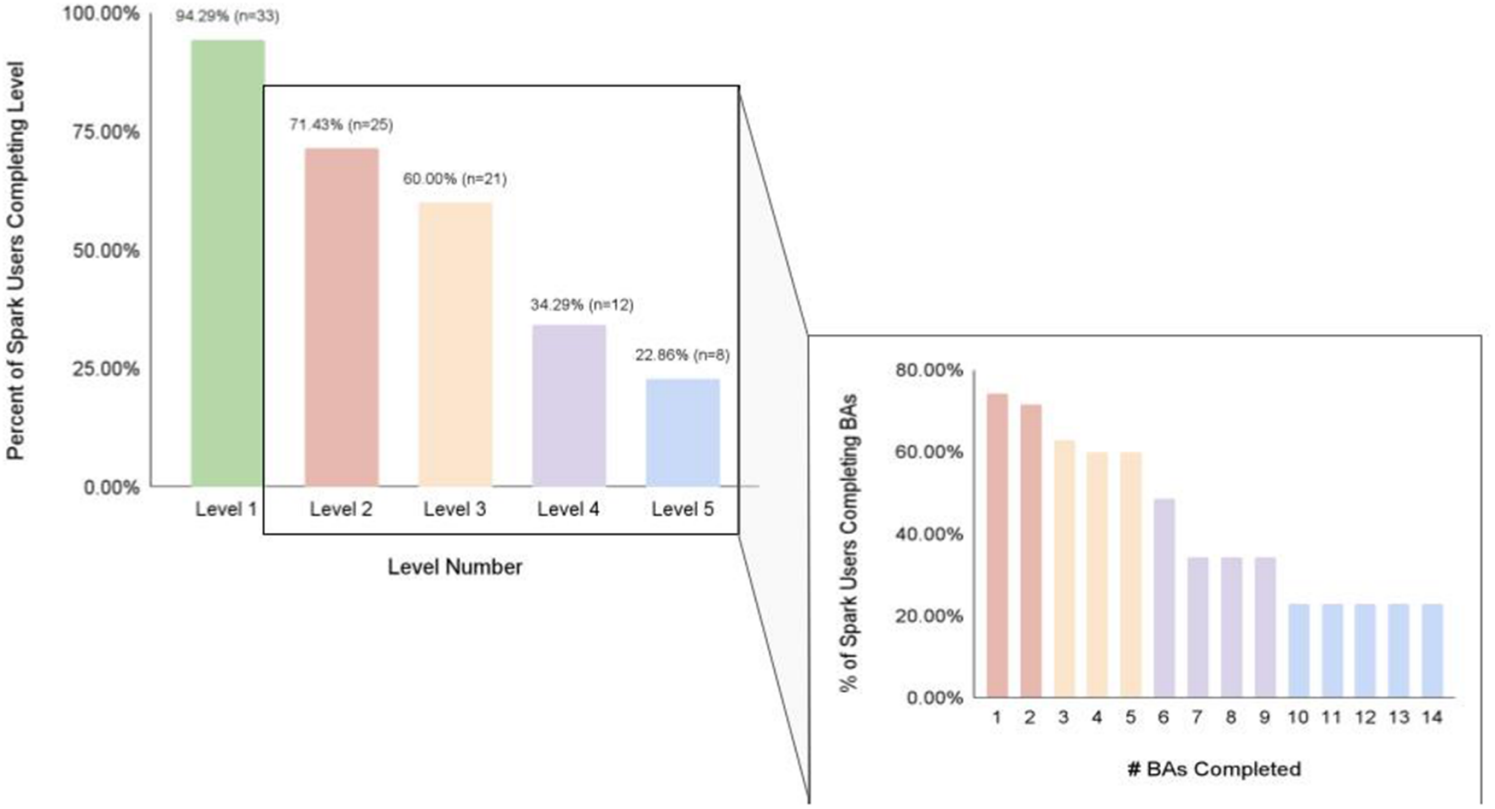
The percent of Spark users completing each level of the Spark intervention, along with the number of behavioral activations completed by a certain percentage of Spark users.

**Figure 5:**
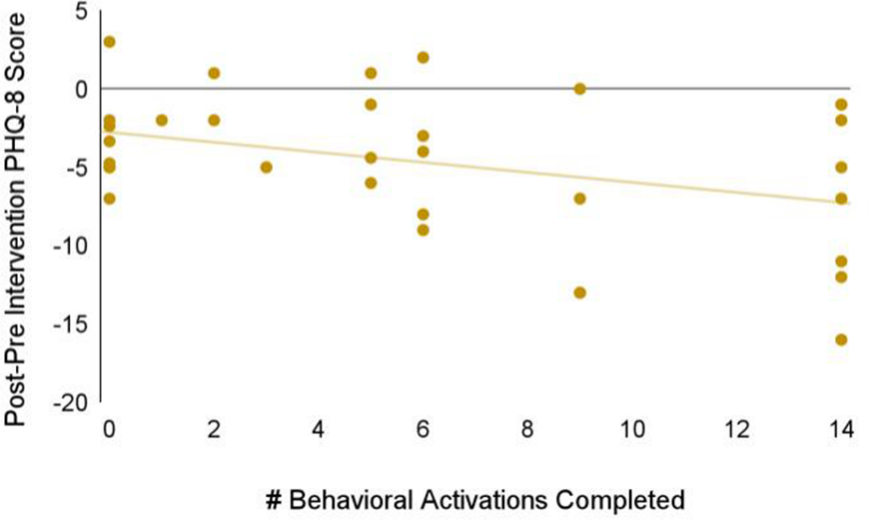
Relationship between the magnitude of change in PHQ-8 scores between the baseline and post-intervention timepoints, and the number of BAs that were completed.

We also investigated app engagement metrics. The median number of daily active users on a given day across the 5-week intervention period was 29%, and the 35-day retention rate was 26% (Figure 3). 94% of participants who received Spark completed level 1, with decreases in level completion in subsequent levels to 23% completing level 5 (Figure 4). Only levels 2–5 consisted of completing behavioral activations. 60% of the participants completed at least 5 behavioral activations (Figure 4). Furthermore, we found a significant negative relationship between the magnitude of change in PHQ-8 scores from the post-intervention and baseline timepoints, and the number of BAs that were completed (*r*(32) = −0.38, *p* = 0.03; Figure 5).

### 3.3. Study safety protocol feasibility

**Figure 6:**
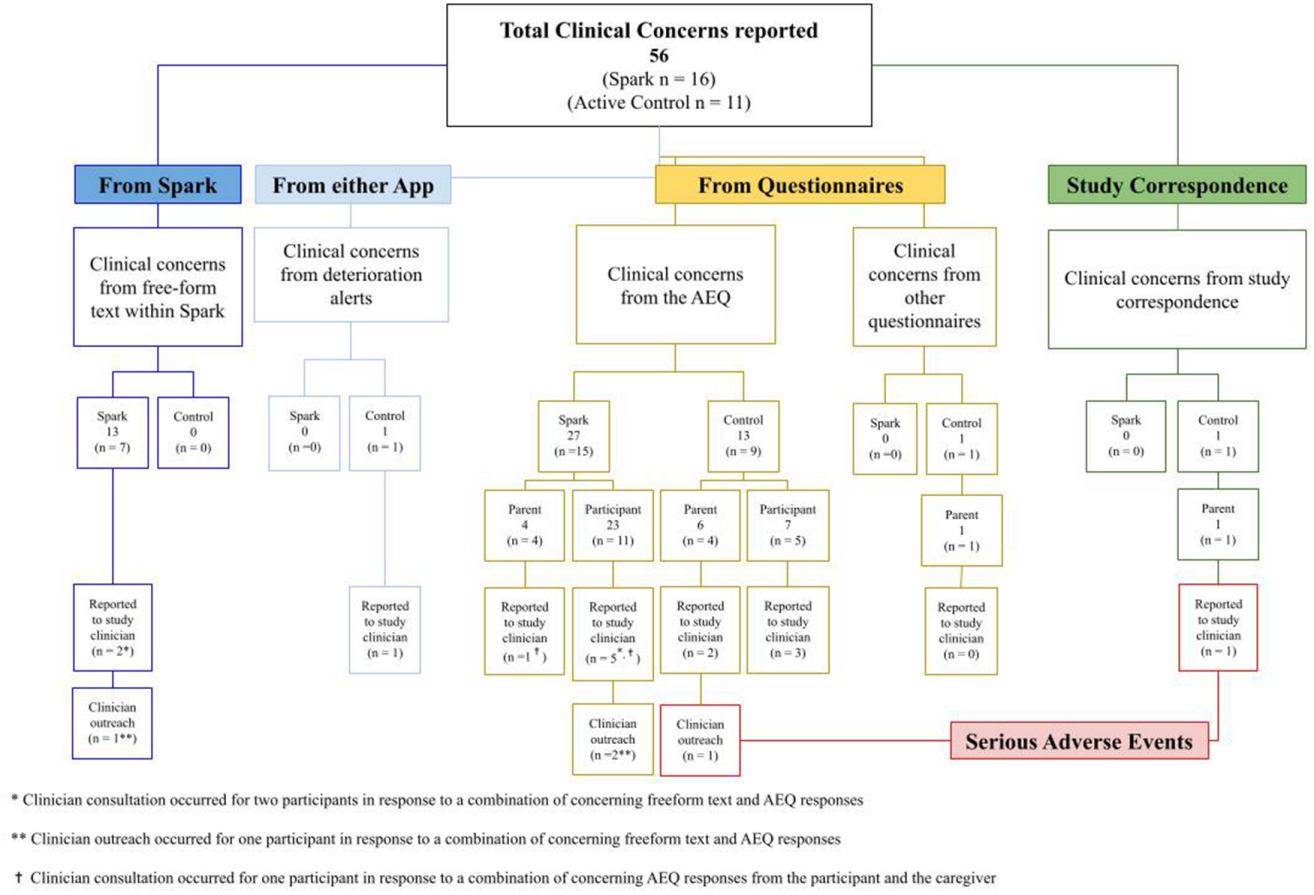
Summary chart of clinical concerns including the sources of clinical concerns, the number of clinical concerns escalated to the study clinician, and the number of clinical concerns that elicited clinician reachout to the participant.

During the 5-week intervention period, 56 potential clinical concerns were logged and evaluated by study investigators (n_Spark_ = 16, n_Active Control_ = 11; see Figure 6). Any text that mentioned symptoms of depression from more serious (e.g., suicidal ideation) to less serious (e.g., cried all day) was logged for review. Of the 40 potential clinical concerns identified in the Spark group, 13 were identified from free-form text entries in Spark and the remaining 27 were identified in the adverse event questionnaire (AEQ), which prompted participants to indicate worsening, frequency, and intensity of mood. Following guidelines listed in the safety protocol, 35/40 logged events did not meet criteria for a potential safety concern and were consistent with expected day-to-day events or expected symptoms of depression, without an indication of worsening in intensity, frequency, or duration. Therefore, the study investigators consulted with the study clinician regarding five of these participants’ clinical concerns. The study clinician used the study safety protocol and their clinical judgment to determine whether clinician outreach was required. The study clinician decided that two out of these five participants were at sufficient risk and contacted them to confirm their safety. Out of the total 16 potential clinical concerns in the Active Control group, one was from a clinical deterioration in depression symptoms (as measured by the PHQ-8), 13 were reported in the AEQ, one was from text entered by a parent in the post-intervention questionnaire, and one was reported in an email response from a parent. Following the same safety protocol, 6/13 logged events did not meet criteria for a potential safety concern; therefore, the study investigators reported seven participants' clinical concerns in the Active Control group to the study clinician. The study clinician decided that one of these participants was at sufficient risk and contacted them and their legal guardian to confirm safety. In summary, 16 out of 35 participants in the treatment group and 11 out of 25 participants in the control group had potential clinical concerns logged, with some individuals in each group having multiple logs, resulting in higher total log counts than the number of participants. Five participants from the treatment group and seven participants from the control group had potential clinical concerns that were escalated to clinicians for safety evaluation. This resulted in 0 AE/SAE classifications for the treatment group and 2 SAEs for the control group.

### 3.4. App efficacy and safety

Three participants were excluded from efficacy analyses due to having completed only the baseline PHQ-8 (n_Spark_ = 1, n_Active Control_ = 2), wwhich did not allow for imputation of missing data.

Within weekly PHQ-8s, 6.1% were missing. No item-level data were missing. Little's test suggested that data were not missing at random $\left(\chi^{2}(26)=52.886,\;p=.0014\right)$. There were no group differences in missing data $\left(\chi^{2}(5)=0.99,\;p=1.00\right)$.

Analyses investigating differences across Group and Week in the number of days between the completion of the baseline PHQ-8 and each weekly PHQ-8 showed a significant effect of Week, *F* = 2,470.35, *p *< .001, as the number of days since baseline increased for each successive weekly PHQ-8. There was no main effect of Group, *F* = 1.96, *p* = 0.16, nor was there an interaction between Group and Week, *F* = 1.158, *p* = .33, indicating that differences in the timing of completion of PHQ-8s by week did not differ between the two groups.

**Figure 7:**
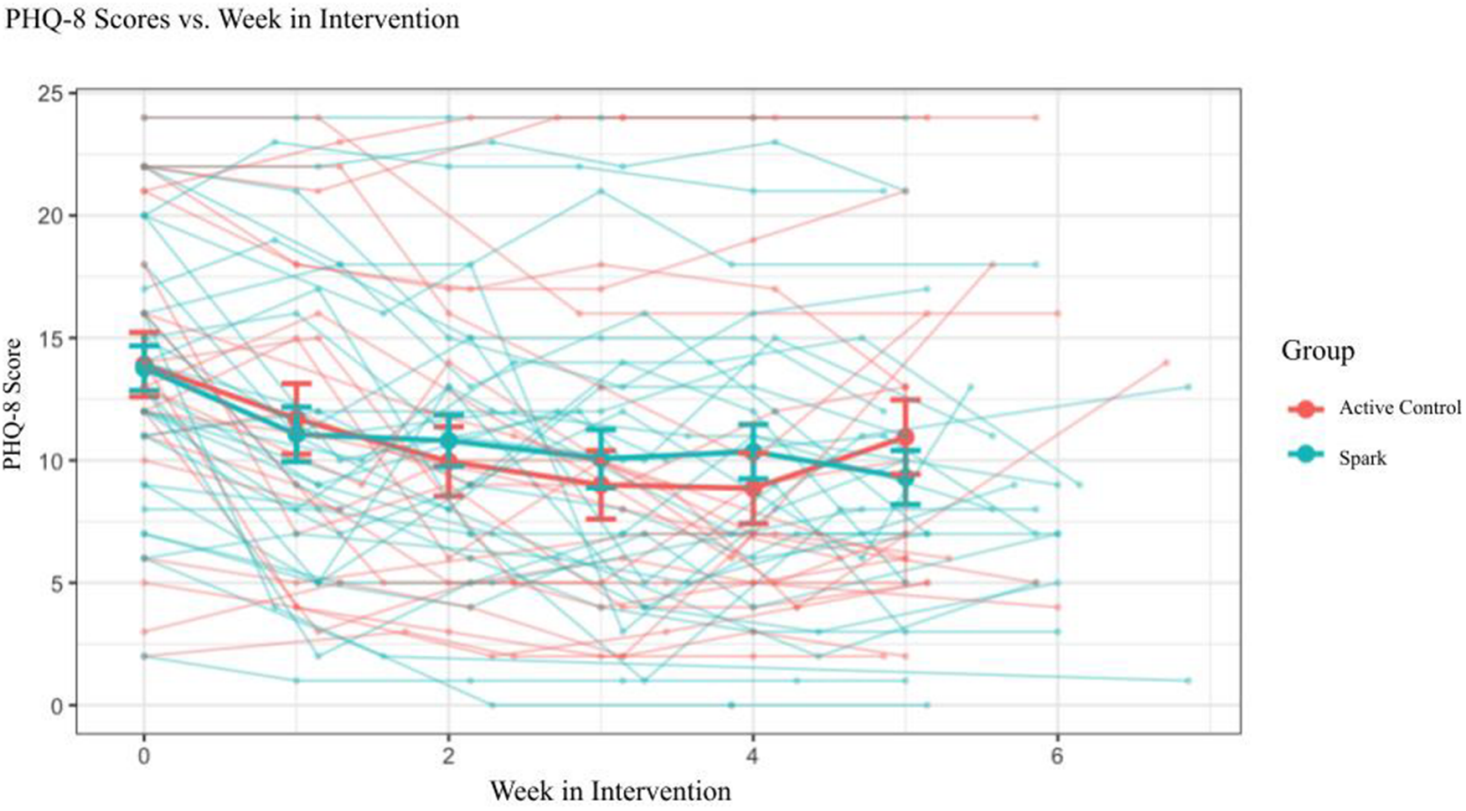
Imputed PHQ-8 scores for participants that completed two or more PHQ-8 questionnaires (*n* = 57) and separated by condition.

The GLMM exploring PHQ-8 scores as a function of Group and Week showed a significant main effect of Week, *F *= 40.600, *p *< .001, demonstrating that depression symptoms declined over time. However, no main effect of Group, *F* = 0.004, *p* = .95, nor Group × Week interaction, *F* = 0.125, *p* = .72, was observed (Figure 7). The lack of a Group × Week interaction appears to have been driven by a larger than expected reduction in symptoms in the Active Control arm, *Δ*PHQ-8_Active Control_ = 3.56, as the average reduction in symptoms in the Spark group, *Δ*PHQ-8_Spark_ = 4.69, was close to reaching a clinically meaningful change (defined as *Δ*PHQ-8 ≥ 5; see Table 6) (46, 76, 77). However, an exploratory analysis showed that Spark users with moderate or higher levels of depression (PHQ-8 ≥ 10) demonstrated, on average, a clinically meaningful reduction in depressive symptoms, while Active control users did not (*Δ*PHQ-8_Spark_ = 5.62 (4.68), n_Spark_ = 26; *Δ*PHQ-8_Active Control_ = 3.72 (5.01), n_Active Control_ = 19).

**Table 6:** Change in depressive symptoms at baseline vs. Post-Intervention by group as evaluated by the PHQ-8.

	Baseline	Post-intervention	Mean difference
Spark, M (SD)	13.76 (5.31)	9.06 (5.76)	4.69 (4.53)
Active Control, M (SD)	13.91 (6.30)	10.36 (6.98)	3.56 (5.03)

In relation to app safety, there were a total of 2 SAEs, which both occurred in the Active Control group. One SAE was reported in the weekly AEQ; a parent reported that their child was hospitalized due to depressive symptoms. The clinician contacted the participant and parent and confirmed the participant was safe and eligible to continue with the study. The second SAE was reported *via* email; a parent wrote that their child had been hospitalized for a suicide attempt. Since the individual was receiving care at the hospital, there was no study clinician reachout. This participant was also withdrawn from the study due to our inability to accurately monitor their safety during the intervention period as they did not complete the AEQ questionnaire over two consecutive weeks during the 5-week intervention period). There were no ADEs or UADEs reported in either group.

No significant effect was determined when comparing baseline and post-intervention mean scores across groups was determined for any other measure (GAD-7, MFQ, PROMIS Pediatric Global Health), except for the MFQ (see Table 7).

**Table 7:** Change in GAD-7, MFQ, and PROMIS pediatric at baseline and post-intervention, mean difference and Cohen's D.

GAD-7				
	Baseline	Post-intervention	Mean Difference	Effect size
Spark, M (SD)	11.26, 35 (4.85)	8.77, 30 (5.98)	−2.49	d = −.18 95% CI [−.58,.18]
Active Control, M (SD)	12.08, 25 (5.20)	10.10, 21 (5.96)	−1.98	
MFQ				
	Baseline	Post-intervention	Mean Difference	Effect size
Spark, M (SD)	18.63, 16 (4.32)	9.00, 10 (6.57)	−4.31	d = .25 95% CI [−.35,.85]
Active Control, M (SD)	12.08, 13 (5.78)	8.00, 8 (4.63)	−4.08	
PROMIS Pediatric (Global Health)				
	Baseline	Post-intervention	Mean Difference	Effect size
Spark, M (SD)	35.88, 35 (6.62)	37.97, 30 (7.86)	2.09	d = .06 95% CI [−.68,.82]
Active Control, M (SD)	34.83, 25 (6.27)	35.50, 21 (6.77)	.67	

## 4. Discussion

The results of this study determined that 1) it was feasible to evaluate a 5-week, self-guided CBT-based mobile app program compared to an active educational control app for an adjunct treatment of adolescents with symptoms of depression in a nationwide virtual and decentralized RCT, 2) adolescents found the app acceptable, and 3) our safety protocols were robust for monitoring participant safety. Additionally, there was a promising reduction in depression symptoms for participants who received Spark, though the difference in symptom reduction between Spark and Active Control was not statistically significant. Finally, there were 0 serious adverse events in the Spark group and 2 serious adverse events in the control group. This suggests that participants in the Spark group were not at greater risk of a serious adverse event than participants in the active control group.

### 4.1. Study feasibility

The enrolled sample successfully represented a range in age, gender, race, ethnicity, and depression symptom severity. Though females were more heavily represented, this is consistent with the etiology of depression in adolescents (78). The recruited sample was racially diverse compared to other feasibility studies, which may have been a benefit of our decentralized approach to virtually recruiting participants nationwide (79). The racial and ethnic background of participants in the study was in line with national racial and ethnic census data and with the demographic distribution of depression among adolescents (80–82). The diversity reflected in the study sample is a strength and may allow for greater generalizability of feasibility, engagement, and usability findings to the wider population of adolescents with depression.

Target enrollment was reached in two months for this trial, demonstrating the success of our online recruitment strategy and the perceived feasibility of our enrollment procedures. This recruitment speed may also underscore the demand for mental health resources in this population and during the COVID-19 pandemic, as well as reflect an interest in and receptivity to digital health solutions. Additionally, our recruitment, enrollment and retention rates were high compared to other feasibility studies that enrolled similar populations (those with depression (83) and/or adolescents (84) through online recruitment for remote interventions (83–85). For example, our enrollment rate was double a feasibility trial evaluating the effectiveness of clinical trials conducted in a virtual setting, or 21% (205 out of 958) vs. 40% (60 out of 150) of participants screened vs. those that enrolled (85). Despite this success, a few areas of improvement were identified. Improvements to increase retention could include sending more regular reminders to participants to remind them to complete questionnaires and additional modalities for reminders, such as text and email notifications. Additionally, tailoring availability of onboarding sessions to later hours in the day or weekends could allow faster enrollment, especially for participants under the age of 18, given the required involvement of legal guardians and scheduling constraints around school hours.

### 4.2. App acceptability

Participants that used Spark rated it as more usable than those that used the Active Control app in terms of enjoyment and in terms of its impact on improving their symptoms of depression. Furthermore, both users of Spark and the Active control rated the app as well above average usability (64). While there was no significant difference in the ratings of usability of the two apps, Spark users rated it, on average, as more usable on the SUS scale than Active Control users, suggesting that its interactive features are easy to use. Engagement was also high for the Spark group: with an engagement rating above 3.5 (out of 5), this is comparable to similar studies (86, 87). All users except one gave Spark an engagement rating above 3 and Spark was rated as significantly more engaging than the Active Control app. Together, this suggests that Spark is highly acceptable to study participants.

App engagement metrics are as good or better than other depression apps on the market. Baumel and colleagues report that the median daily open rate for real-world usage of depression apps is 4.8% (88), and is 4.06 times higher for research studies (88, 89), which is lower than the median daily active use we found. They also found that the 30-day retention rate is 3.3% for real-world usage of mental health apps (88). Even a 4.06 fold increase in average engagement for apps in research studies (89) would put our 35-day retention rate of 26% above the average. Though adherence (completion of all levels in the app) was only at 23%, engagement in digital therapeutics for mental health is a challenge across the field (90). This low adherence may be contributing to the non-significant difference in changes in PHQ between groups. Interestingly, the relationship between the number of behavioral activations completed and the reduction in PHQ-8 scores is similar to or stronger than other studies that report little or no relationship between app dose and treatment response (91–93). This suggests that if engagement increases, this may facilitate even greater improvements in depression symptoms.

One reason Spark may have had high engagement is because of its reliance on BA, which is inherently self-paced and may appeal to self-motivated adolescents. A 2021 meta-analysis of digital intervention studies showed that flexibility was a component often used to increase adherence and engagement (36). Furthermore, users of apps that help treat depression have stated a desire to have space for positive emotions within digital mental health products they are using (94), a quality inherent to BAs. However, for individuals who may not feel self-motivated, it is important to incorporate additional features to enhance engagement, like reminders. The therapeutic qualities of BA can be further enhanced in the digital setting with the inclusion of additional features allowing for increased personalization, gamification, and ease of use (36), which will be important for future versions of Spark.

It is worth noting that operationalizing and measuring meaningful engagement is a challenge in the field of digital therapeutics and is critical for understanding how adherence and engagement impacts therapeutic outcomes (94). This is an area in which Limbix is actively working (90). In future versions of Spark, a focus on improvement engagement, like including mood-tracking activities, mindfulness, psychoeducation, and relapse prevention in addition to the behavioral activation activity scheduling that was included here may help to improve outcomes. Furthermore, though each level could have taken up to 60 min to complete, which may seem like too long for adolescents to be able to engage, we do not believe that this was actually a barrier to engagement. This time was purposely overestimated so that teens would not feel discouraged if it took longer to complete a module than anticipated. This estimate also included time to do BA activities outside of the app, and additionally, adolescents could go at their own pace, using the app for only a few minutes per day, and still complete each module. We felt it was important to keep this amount of content in the treatment so that we could retain essential clinical components to improve outcomes; having an evidence-based treatment is rated as one of the five critical features of evaluating mental health apps according to the American Psychiatric Association (96) and is viewed as an increasingly necessary feature of digital health solutions (97). Therefore, we believe the primary goal is to modify the app to make the material more engaging while still maintaining a high standard for clinical quality. Though these are preliminary analyses, these results suggest promising directions for future work.

### 4.3. Study safety protocol feasibility

A third aim of this study was to develop and test the feasibility of using a detailed, thorough method for monitoring safety in a decentralized, virtual trial of a mobile application. Typically, safety protocols for studies of digital interventions are either not reported (95, 96) or consist of unstructured monitoring with safety intervention at the investigator's discretion (97). Nevertheless, a thorough approach as implemented here may be especially critical for ensuring safety of study participants within the context of a completely virtual and decentralized trial. Additionally, the use of mobile technology affords the opportunity to standardize data collection around safety rather than relying exclusively on spontaneous reporting. The safety protocol was successful in ensuring participant safety throughout the study period. It provided a standardized and rigorous method to track participant and guardian reported clinical concerns in both study arms. This protocol allowed study investigators to determine which clinical concerns met criteria to be considered adverse events as well as the severity of such events. The clinician outreach approach outlined in the protocol was feasible and effective for determining relatedness of adverse events to the study apps and assuring participant safety. Opportunities for refining the safety protocol in the future could include increasing automation in identifying potential clinical concerns to reduce the potential for human error or oversight.

### 4.4. Preliminary App efficacy & safety

The preliminary clinical efficacy and safety of Spark was evaluated compared to an active control condition. The lack of serious adverse events in the Spark group, compared to two in the Active Control group, suggests that Spark does not pose any additional risk to users. Efficacy was measured by a reduction in depressive symptoms as measured by the PHQ-8. There was a significant main effect of Time, indicating that both groups reported improvements in symptoms of depression over the intervention period. While we did not observe a statistically significant difference in symptom reduction between groups, Spark users experienced a greater numeric decrease in PHQ-8 scores compared to Active Control users. The reduction of depression symptoms in the Spark group was promising, as the average reduction in depression symptoms approached a clinically meaningful change. Symptom reduction in the Spark group may have been limited by a floor effect introduced by the inclusion of participants with all levels of baseline symptom severity. This possibility was supported by a *post hoc* analysis of only participants with at least moderate baseline symptom severity that showed a clinically meaningful reduction in symptom severity at post-intervention. In fact, recent evidence suggests that digital interventions may be most effective for more severe forms of depression (98).

The lack of statistical significance in symptom reduction between groups is not surprising, given that this trial was not designed or powered to detect statistical differences in symptom reduction between Spark and the Active Control. Notably, this finding seemed to have been driven at least in part by a larger than expected reduction in symptoms in the Active Control group (26, 99–101), which might be explained by a number of study considerations. First, the study design did not control for participants beginning new treatment or changing treatment for a mental health condition immediately prior to or during the study intervention period. Additionally, the psychoeducational material in the Active Control app may have had therapeutic impact, as psychoeducation is used as a form of treatment (102) and is considered a therapeutic element of CBT. Finally, changing impacts of the pandemic may have played a role, as changes to federal, state, and regional policies occurred, including those related to remote schooling, during the conduct of the trial. Future studies powered to detect statistical differences between groups will be necessary to evaluate the efficacy of Spark relative to an active control condition.

### 4.5. Limitations and future directions

Though these data support study feasibility and the acceptability of the Spark app, limitations remain. The recruited sample was predominantly female (78, 103), which is consistent with prevalence rates of depression in adolescence (104). However, a limitation is that these results are not generalizable to males and gender non-binary individuals. Future studies should consider alternative sampling methods that result in a more equal sampling to better understand the effects in non-female populations. In addition to this, our eligibility criteria required that participants were under the care of a US-based primary care and/or licensed mental healthcare provider. This criteria was included to; 1) evaluate the feasibility of the Spark app as an adjunct treatment for depression and 2) manage participant safety. We acknowledge that many adolescents are not under the care of a US-based primary care and/or licensed mental healthcare provider and as a result, our sample may not be generalizable to the adolescent population in the US. Participants and their legal guardians were required to be fluent in English in order to enroll in the study and use the study apps, in turn limiting access for those who are not English-speaking. While for this study no participants were determined ineligible due to this criteria, individuals from minority populations who do not speak English are in need of mental health services and future work will be needed to determine whether it is feasible to use Spark in such populations. Also, we included participants that were receiving other forms of treatment at baseline. Though excluding such participants may have increased the efficacy of Spark, this choice was made because Spark is intended to be used as an adjunct treatment and we wanted to make Spark widely available to those who were looking for additional resources during the COVID-19 pandemic. This likely increased the ecological validity of this study given Spark's intended use. Because efficacy analyses were preliminary, we did not statistically control for changes in treatment for mental health conditions prior to, or during the study intervention period or stratify this variable between groups. As a result, reductions in depression severity or lack of group differences could be attributed to changes in concomitant treatments that participants were receiving. Future studies should ensure stability on concurrent treatments and control for changes in treatment during the study intervention period. Engagement analyses were limited to subjective measures, whereas objective measures of app use analytics would provide a more complete picture of engagement. Additionally, while the study's safety protocol was supported based on AE ratings and clinical concern rates, improvements can be made. In this study's safety protocol, we withdrew participants from the study if they did not complete two weekly questionnaires in a row. This criterion was implemented in order to motivate participant completion of questionnaires, including the AEQ, which would allow better monitoring of participant safety. For future studies, it would be preferable to maintain participant involvement in the study and remove this criteria for withdrawing participants, in order to not miss potential data from these withdrawn participants. An additional limitation of study procedures was that suicidality and comorbidities were not assessed using standardized measures in every participant to confirm eligibility. While thorough screening measures were taken to provide the participants with a self-reported confirmation of eligibility, in future studies, we may implement standardized screenings.

Lastly, we recognize that this study was not powered to detect statistical differences between groups and all statistical analyses are considered exploratory. Future studies will be required to evaluate efficacy, safety, and engagement of Spark relative to an active control condition or other digital therapeutics.

### 4.6. Conclusion

This feasibility study demonstrated the robustness of online recruitment techniques, strong engagement with and potential therapeutic benefit of Spark, and the effectiveness of the novel safety protocol to monitor and ensure patient safety. These findings will be used to inform and direct future product development as well as a powered RCT to evaluate app efficacy. The results of this feasibility trial provide preliminary support for the use of Spark as a novel digital treatment for adolescent depression and may point to the utility of digital therapeutics in addressing existing barriers in access to effective mental health care.

## Data Availability

The raw data supporting the conclusions of this article will be made available by the authors, without undue reservation.
