# Supplementary material for: A CBT-based mobile intervention as an adjunct treatment for adolescents with symptoms of depression: a virtual randomized controlled feasibility trial
Source: Front Digit Health. 2023 May 23;5:1062471. doi: 10.3389/fdgth.2023.1062471 (PMC10262850; doi:10.3389/fdgth.2023.1062471)
Supplement: Supplementary file 1 [file Table1.docx]

**Supplemental Materials**

1. **Additional Measures**

**COVID-19 Questionnaire**

At baseline, participants and consenting guardians were asked to evaluate the impact of the COVID-19 pandemic on their lives. A series of phrases were presented from which the participant could select any number of answers that described both positive and negative impacts of the pandemic, in addition to the option of free-form text response. This questionnaire was presented as a subset of questions located within the baseline and post-program questionnaires. This assessment was delivered during baseline and post-intervention.
